# Effects of the COVID-19 Pandemic on Early Childhood Development and Mental Health: A Systematic Review and Meta-Analysis of Comparative Studies

**DOI:** 10.3390/psycholint6040062

**Published:** 2024-11-25

**Authors:** Sasha Alcon, Sa Shen, Hong-nei Wong, Cynthia R. Rovnaghi, Leni Truong, Jordan K. H. Vedelli, Kanwaljeet J. S. Anand

**Affiliations:** 1 Department of Pediatrics, Stanford University School of Medicine, Stanford, CA 94304, USA; 2 Stanford Child Wellness Lab, Maternal & Child Health Research Institute, Stanford Cancer Institute, Stanford University, Stanford, CA 94305, USA; 3 Quantitative Sciences Unit, Stanford University School of Medicine, Stanford, CA 94304, USA; 4 Medical Education Librarian, Lane Medical Library, Stanford University School of Medicine, Stanford, CA 94304, USA; 5 Undergraduate Program, Stanford University School of Humanities & Sciences, Stanford, CA 94309, USA; 6 Department of Anesthesiology, Perioperative, and Pain Medicine, Stanford University School of Medicine, Stanford, CA 94309, USA

**Keywords:** behavioral health, coronavirus, young children, quarantine, developmental health

## Abstract

From 2020 to 2023, the coronavirus-19 (COVID-19) pandemic exposed children to a variety of adverse childhood events, including parental loss, abuse, and disruption in services, and it exacerbated societal inequities. Studies evaluating the mental health of older children and adolescents reported increases in depression and anxiety symptoms, but no reviews have addressed the effects of the pandemic on preschool children. This systematic review and meta-analysis is the first to explore these effects. The goal was to analyze and synthesize longitudinal cohort studies to determine impact of the COVID-19 pandemic on the development and mental health of young children. Searches of multiple databases were performed for studies published between 2018 and 2023 with pre- and post-pandemic evaluations of the mental health or development of preschool children (aged 0–6 years) using objective measures and according to Preferred Reporting Items for Systematic Reviews and Meta-Analysis (PRISMA) guidelines. Cohen’s d effect sizes were calculated for each study that utilized the Strengths and Difficulties Questionnaire (SDQ), Ages and Stages Questionnaire (ASQ), or the Child Behavior Checklist (CBCL). Random-effects mixed models combined the estimates of effect sizes to calculate the overall mean effect size. The meta-analyses included 22,348 children from 16 countries. The analyses showed small increases in emotional symptoms and conduct problems, as well as increases in emotional reactivity, anxiety/depression, withdrawal symptoms, attention problems, and aggressive behaviors. A decrease in fine motor and personal–social skills was noted. Studies not included in these meta-analyses also showed negative effects on language and executive function. This systematic review characterizes the detrimental effects of the COVID-19 pandemic on the mental health and development of preschool children across the world. Our results suggest the vulnerability of early childhood to pandemic-related disruptions, although the heterogeneity in study design and child characteristics may limit some of these conclusions.

## 1. Introduction

Children experienced stressors and significant environmental disruptions during the pandemic. School closures disrupted mental health counseling, early intervention programs, access to peers, group activities, and safe spaces [1,2]. In the United States, approximately 56% of childcare centers temporarily closed or shut down indefinitely during the pandemic [3]. Preschool children aged 0–6 years are at greatest risk for child abuse and neglect [4,5], but they may not report these adverse experiences because of their innate behavioral inhibitions and limited verbal repertoire [6]. Because the youngest children are completely dependent on their parents, Adverse Childhood Experiences (ACEs) in early life are underreported and underestimated [4,5,7,8]. In addition to ACEs or other stressors [9], preschool children are exposed to frequent, low-grade Early Life Stress (ELS) due to parental absence [10] or incarceration [11,12], high parenting stress [13,14], parental ACEs [15], inconsistent and variable parenting, low parental self-esteem, and/or low resilience [16–18]. Increased screen time for children and parents has further reduced their opportunities for supportive parenting and positive and dyadic interactions [19–22], leading to the inadequate buffering of chronic stress in small children [23,24].

Preschools and daycare centers serve as critical resources in which children develop social skills through peer interactions and structured learning activities. Additionally, multiple studies have shown childhood social isolation is associated with poor school performance, unhealthy relationships, and criminal behavior as children grow up, at times lasting even into adulthood [3,25–30]. This loss of learning spaces and social interactions was further exacerbated by difficult home learning environments that were impacted by the pandemic.

Many parents faced significant impacts on employment, including needing to take leave, reduce work hours, or even leave their jobs to care for their children [31]. This disproportionately affected families from low socioeconomic backgrounds [31]. Furthermore, increased stressors, such as financial stress or changes in living conditions, can negatively impact parental behavior and affect cognitively stimulating parent–child activities, leading to suboptimal home learning environments and long-term effects on child school readiness and school achievement [32,33].

Araujo et al. described the effects of previous pandemics such as H1N1 influenza, acquired immunodeficiency syndrome (AIDS), and Ebola on children, including increased risks of adverse childhood experiences (ACEs), toxic stress, mental/emotional problems, developmental delays, and health problems in adulthood [34]. Given the well-known effects of ACEs and other stressors on the mental health of children [35–39], it is crucial to characterize the effects of the pandemic on early childhood development. Although previous studies have focused on older children and adolescents, this is the first meta-analysis to examine the mental health and development of preschool children. As the mental health crisis evolves, these analyses may help to support policies aimed at increasing capacity for younger children and designing social restrictions to limit their adverse effects on this vulnerable population.

## 2. Materials and Methods

### 2.1. Search Strategy

A systematic review and meta-analyses were conducted following the Preferred Reporting Items for Systematic Reviews and Meta-Analyses (PRISMA) guidelines. This systematic review was registered with the International Prospective Register of Reviews (PROSPERO) (CRD42024536928) in April 2024, after the review was conducted. The review protocol may be viewed on the PROSPERO register. The Covidence^®^ software platform (Melbourne, Australia; https://www.covidence.org/) was used to search for and evaluate relevant studies from Embase, PsycINFO, and PubMed. Both Embase and PubMed are extensive databases that provide over 30 million abstracts and papers and are well regarded as the top medical and healthcare research databases. PsycINFO was chosen given its focus on psychology-related research, which is pertinent to our query. For each database, a variety of MeSH terms were used, including preschool, toddler, infant, development, coronavirus, and mental health (see detailed search strategies in Appendix A). These keywords were entered utilizing Boolean search operators and all searches were conducted from June 2022 to June 2023.

### 2.2. Selection Criteria

All studies were reviewed by two reviewers for title and abstract screening. Studies that met the criteria from the initial screen were then reviewed independently by three reviewers for inclusion. Studies were included if they (1) were published in peer-reviewed journals, (2) included participants aged 0–6 years old, (3) evaluated participants at two time-points, from before (2018–2020) and after the specific country’s COVID-19 quarantine period (2020–2023), (4) contained objective assessments for one or more domains of child development, including physical, cognitive, behavioral, emotional, communicative, and social development, (5) were written in English, and (6) contained data representing 80% or more participants. Children aged 0–6 years of age were chosen as children under the age of 6 were most affected by the closure of preschools and daycare centers. We excluded studies only reporting qualitative data, unpublished preprints, abstracts, case series, and case reports.

### 2.3. Data Extraction

We evaluated the title and abstract of each citation for possible relevance. Full-text versions of those papers deemed possibly relevant were reviewed and included if the study inclusion criteria were met.

**Figure 1.**
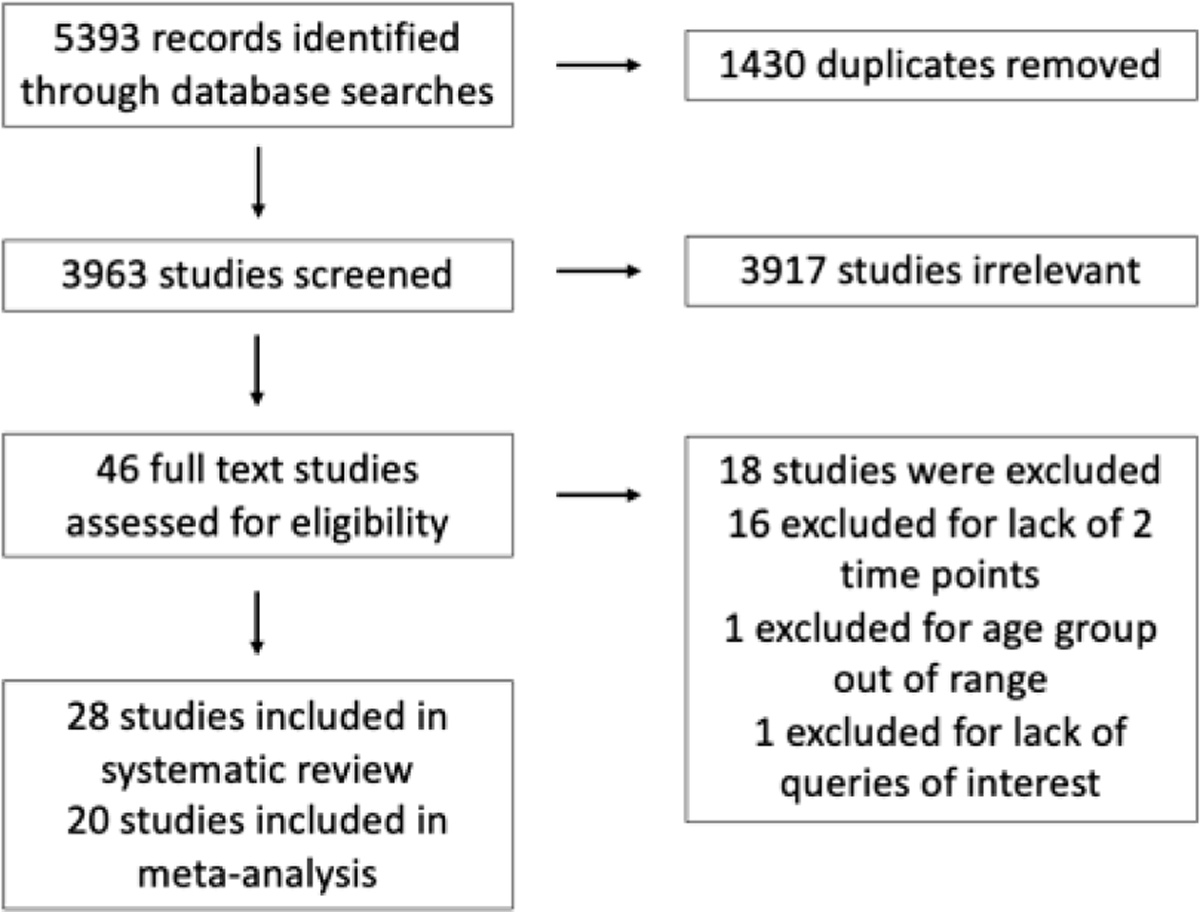
Flow chart detailing the systematic review of the literature.

Our systematic review contained 28 studies, with 20 reports eligible for quantitative meta-analyses based on the outcomes measured (Figure 1).

### 2.4. Study Quality

Quality assessment of the selected studies was performed based on National Institutes of Health (NIH) criteria for observational and cohort studies [40]. All eligible studies meeting our selection criteria, noted above, were scored 0 vs. 1 for each of the 14 quality criteria and summed for a total quality score [40]. We assigned ‘good’ quality for studies scoring 9 or higher, ‘fair’ quality for those with scores of 5 to 8, and ‘poor’ quality for studies scoring 0 to 4. Only studies with ‘good’ or ‘fair’ study quality were included in our meta-analyses.

### 2.5. Statistical Data Analyses

Data on the population demographics, study characteristics, and child outcomes were extracted from each study, databased, and reviewed twice to minimize errors. Given the limited number of studies, data were extracted by a single reviewer, and all data were reviewed by three additional team members independently. If data were missing, authors were contacted via email to obtain additional data.

For each study, differences between the mean pre- and post-pandemic scores were calculated to estimate between-group effect sizes. Under the assumption of independence, Cohen’s d effect sizes were derived from the available descriptive statistics (means, standard deviations, correlation coefficients) or the test statistics (T statistic, F statistic) reported in each study. Effect size and its 95% confidence intervals were estimated for each outcome of interest.

Random-effects mixed models were used to combine the estimates of effect sizes to calculate the overall mean effect size. To evaluate whether the true effect sizes were exchangeable across studies, study ID was specified as a random-effects term in each model. Both the estimated mean effect size and random-effects variance were estimated. Forest plots were used to summarize the effect sizes of individual studies. Each study and combined effect sizes were depicted as point estimates bounded by confidence intervals.

Homogeneity tests were performed to examine the variation in study outcomes across studies. The inconsistency of studies’ effect sizes was measured by Cochran’s Q and I^2^ statistics. Cochran’s Q is the weighted sum of squared differences between individual study effect sizes and the combined effect size across studies, whereas the I² statistic describes the percentage of variation across studies that is due to heterogeneity rather than chance [41].

Publication bias was examined graphically using funnel plots, which plot study size on the vertical axis and effect size on the horizontal axis. Funnel plot asymmetry was examined and tested using four different methods: Egger weighted linear regression [42], Begg Rank Correlation [43], Funnel Plot Regression [44], and Trim and Fill methods [45]. The meta-analyses were performed using SAS v9.4 statistical software (SAS Institute, Cary NC, USA), and a SAS macro (PUB_BIAS) was implemented [46,47]. The critical threshold for α-error was set at 0.05.

## 3. Results

### 3.1. Study Characteristics

The studies eligible for the systemic review ranged in sample size from 40 to 200,000 participants and 22,348 subjects were included in the quantitative meta-analysis. Participants were followed for periods from 3 weeks to over a year.

Eligible studies came from a variety of locations around the globe. Twelve European studies were found, including five from Italy, two from Germany, one that combined children from Austria and Italy, and one each from Spain, Switzerland, the United Kingdom, and Denmark. Five studies came from North America, with three from the US and two from Canada. Three studies were conducted in China, and the remainder were from Turkey (two), Israel (two), Japan (two), Nepal (one), and Brazil (one). The study characteristics are presented in Table 1. Additional study characteristics can be found in Table A1.

Only one study included children with known risk factors for neurodevelopmental delay [48]. Of the studies analyzed, 12 studies used the Strengths and Difficulties Questionnaire (SDQ) to assess 5622 pre-pandemic and 5042 post-pandemic children (*n* = 10,664), five studies used the Child Behavior Checklist (CBCL) to evaluate 3262 pre-pandemic and 3899 post-pandemic children (*n* = 7161), whereas three studies used the Ages and Stages Questionnaire (ASQ-3) to assess 3033 pre-pandemic and 989 post-pandemic subjects (*n* = 4022). The remaining studies used a variety of other measures including the Oxford Communicative Development Inventory (O-CDI), the Spence Preschool Anxiety Scale (PAS), Griffiths Scale for Childhood Development (GCSD), the Child Faces Task, and new tools for measuring anxiety or depression.

### 3.2. Changes in Strengths and Difficulties Questionnaire (SDQ)

**Figure 2.**
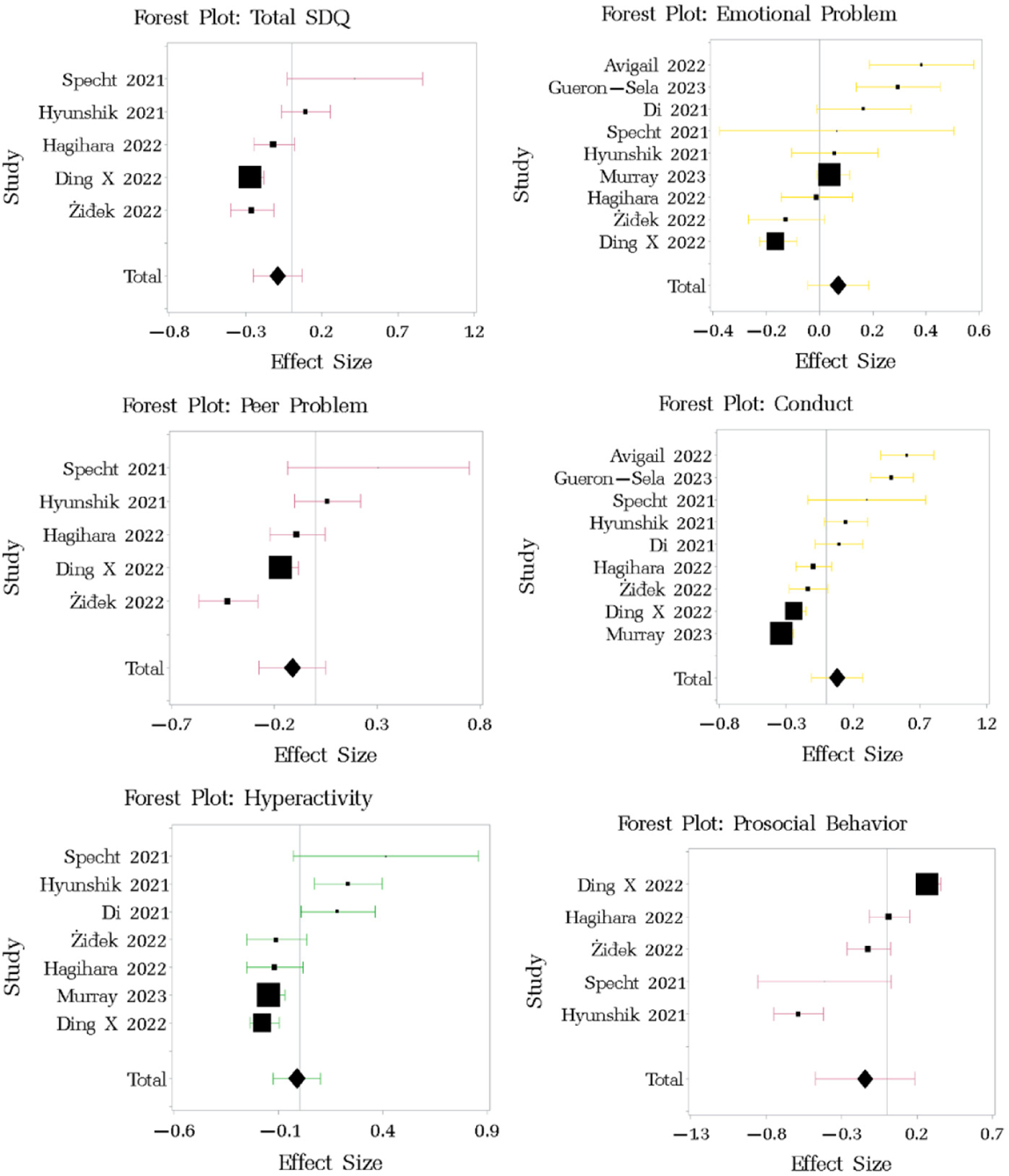
Forest plot of changes in SDQ among children pre- and post-pandemic. Effect size of each study is presented in square. Overall effect size across all studies is presented in diamond. Studies referenced in this figure: [48,52,56–58,61–65,70,74].

**Figure A1.**
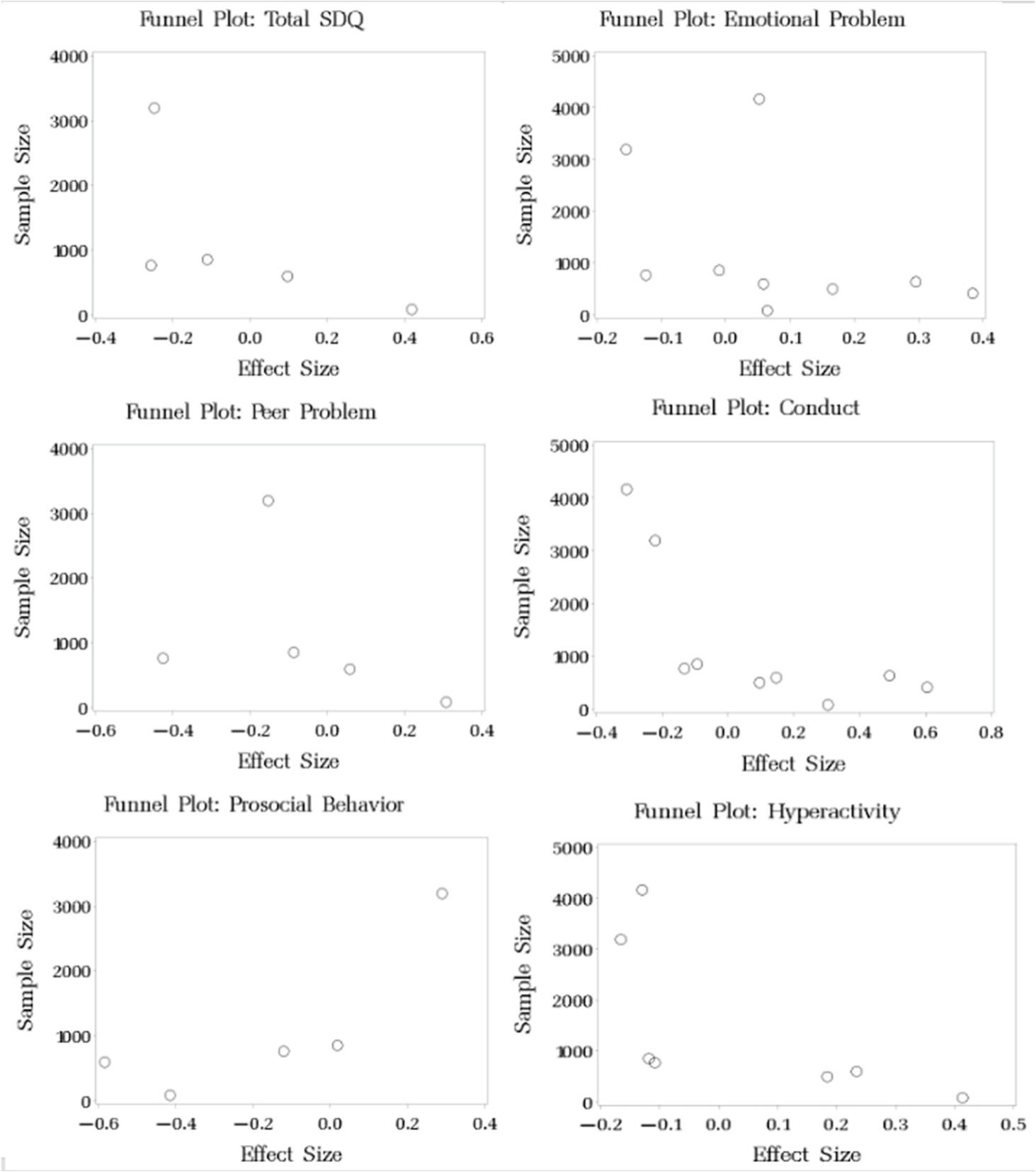
Funnel plot of studies examining changes in SDQ pre- and post-pandemic.

The forest plot for the effect size of pre- and post-pandemic changes in SDQ (Figure 2) revealed minor decreases in the total score (d = −0.088, 95% CI −0.247 to 0.07), prosocial behaviors (d = −0.145, 95% CI −0.474 to 0.185), hyperactivity (d = −0.012, 95% CI −0.124 to 0.1), and peer problems (d = −0.112, 95% CI −0.275 to 0.501), while emotional symptoms (d = 0.07, 95% CI −0.044 to 0.184) and conduct problems showed a negligible increase (d = 0.081, 95% CI −0.111 to 0.273) (Table 2). No publication bias was evident from the funnel plot (Table A2, Figure A1, Appendix B). Moderate or high inconsistency in the study results occurred across the SDQ subscales and total SDQ scores (I^2^ range: 54.17% to 93.57%). Cochran’s homogeneity Q tests suggested significant heterogeneity across the studies and in their outcomes (Table A3, Appendix B), although the between-study variance was small across the total SDQ and subscale scores.

### 3.3. Changes in Ages and Stages Questionnaire (ASQ-3)

**Figure 3.**
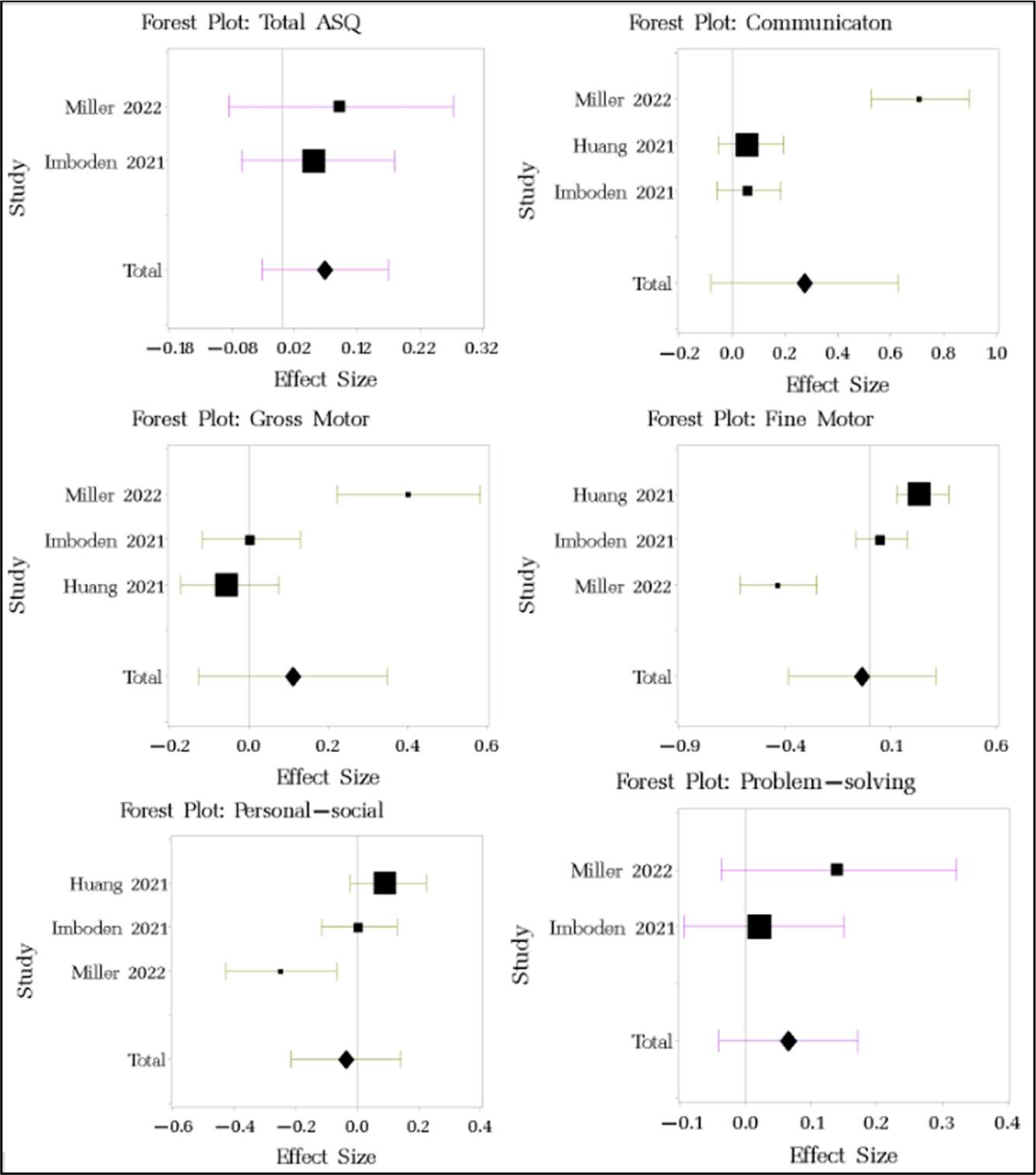
Forest plot of changes in ASQ-3 among children pre- and post-pandemic. Studies referenced in this figure: [47,50,69].

**Figure A2.**
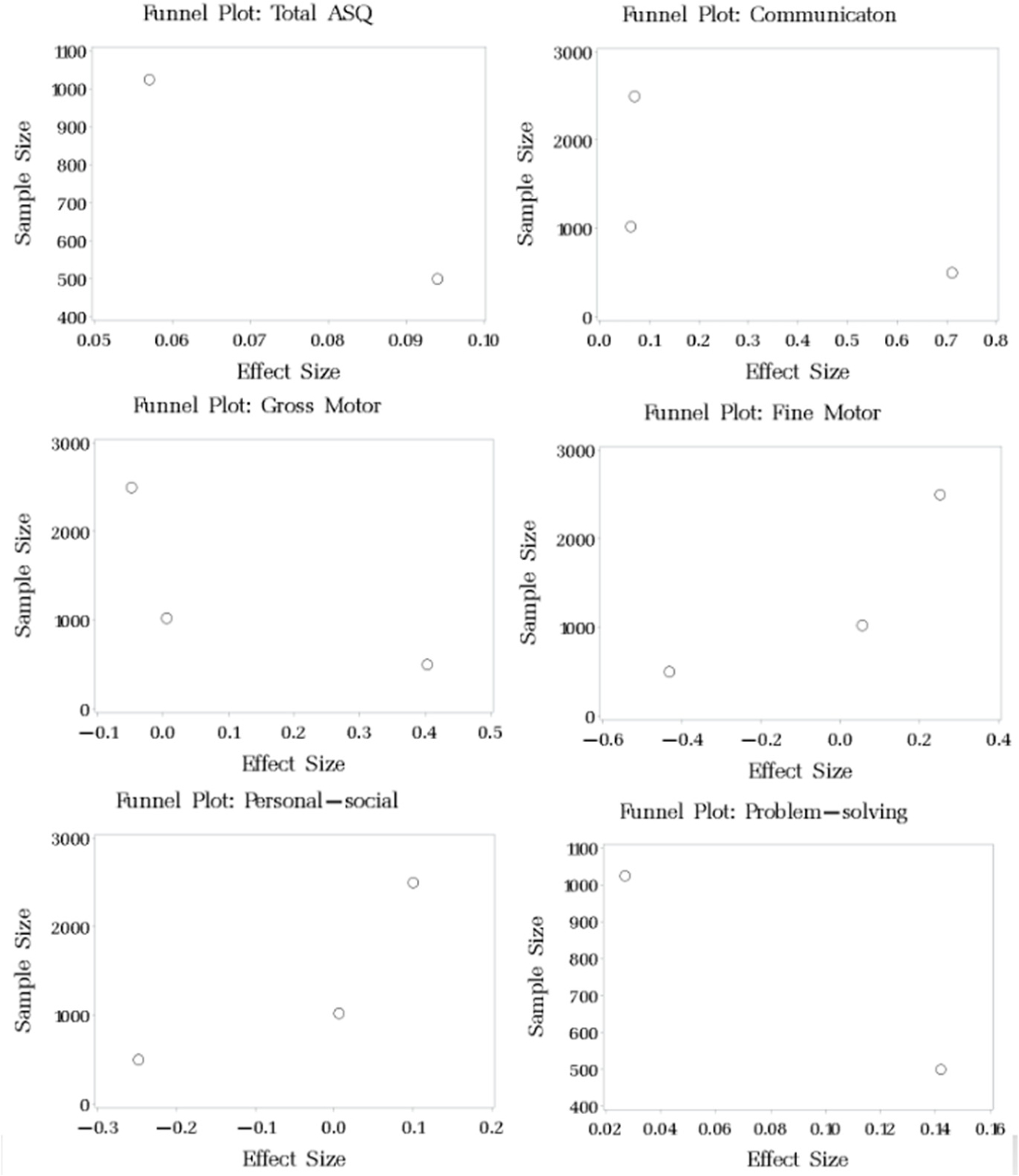
Funnel plot of studies examining changes in ASQ3 pre- and post-pandemic.

The effect sizes of the changes in ASQ-3 scores pre- vs. post-pandemic (Figure 3) showed negligible-to-small improvements in communication (d = 0.274, 95% CI −0.08 to 0.628), gross motor (d = 0.111, 95% CI −0.127 to 0.349), problem-solving (d = 0.065, 95% CI −0.041 to 0.171), and overall scores (d = 0.069, 95% CI −0.032 to 0.17), coupled with minimal decreases in fine motor (d = −0.036, 95% CI −0.384 to 0.312) and personal–social skills (d = −0.036, 95% CI −0.215 to 0.143) (Table 2). The funnel plot showed no evidence of systematic publication bias (Table A4, Figure A2, Appendix B). The χ^2^-test for homogeneity was significant for communication, gross motor, fine motor, and personal–social scores, suggesting significant heterogeneity across the studies (I^2^ values > 80%). The total ASQ and problem-solving skills indicated no significant heterogeneity across the studies. The between-study variance was small for the total ASQ score and all the subscales (Table A5, Appendix B).

### 3.4. Changes in the Child Behavior Checklist (CBCL)

**Figure 4.**
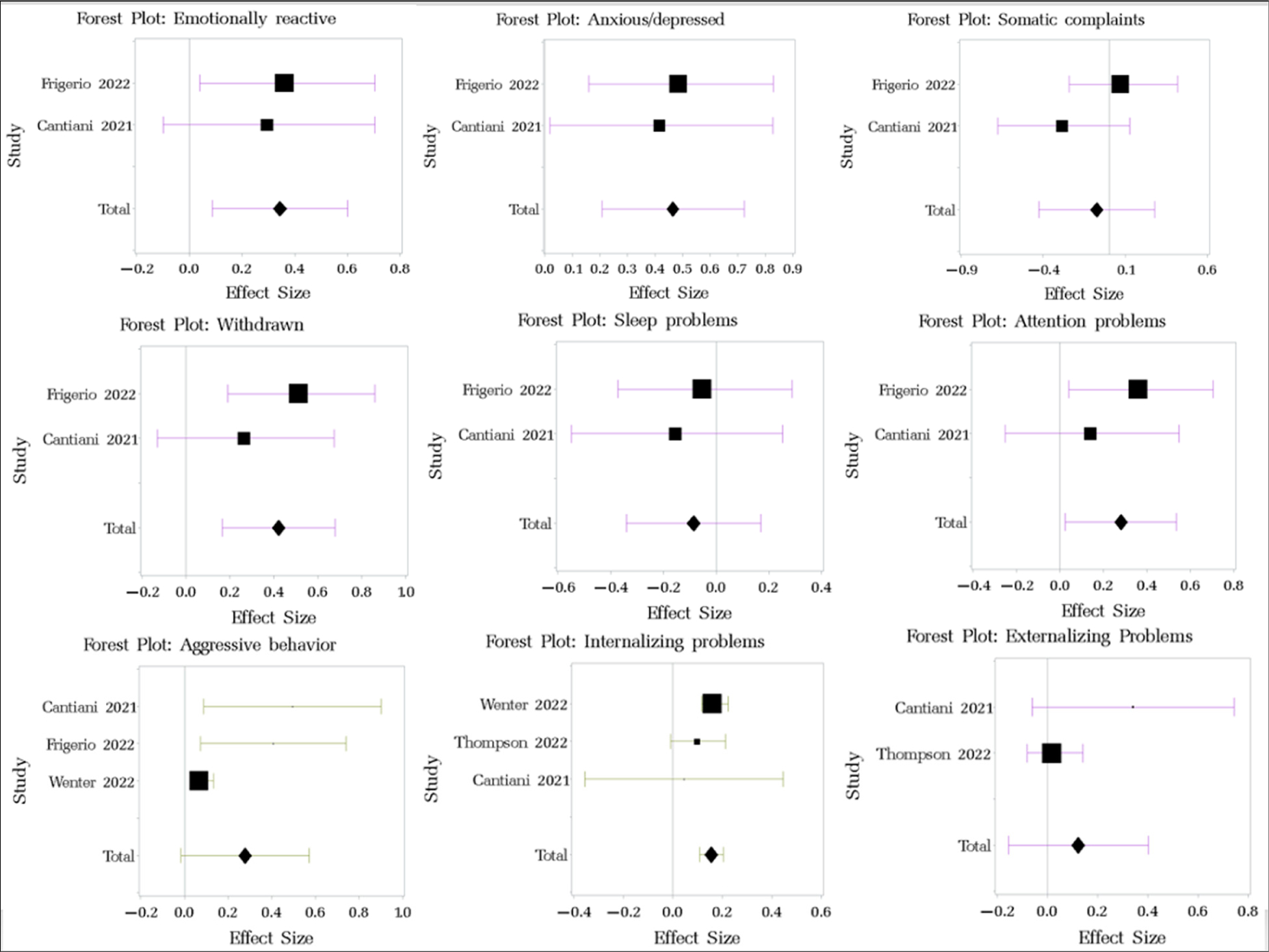
Forest plot of changes in CBCL among children pre- and post-pandemic. Studies referenced in this figure: [49,54,60,66,67].

**Figure A3.**
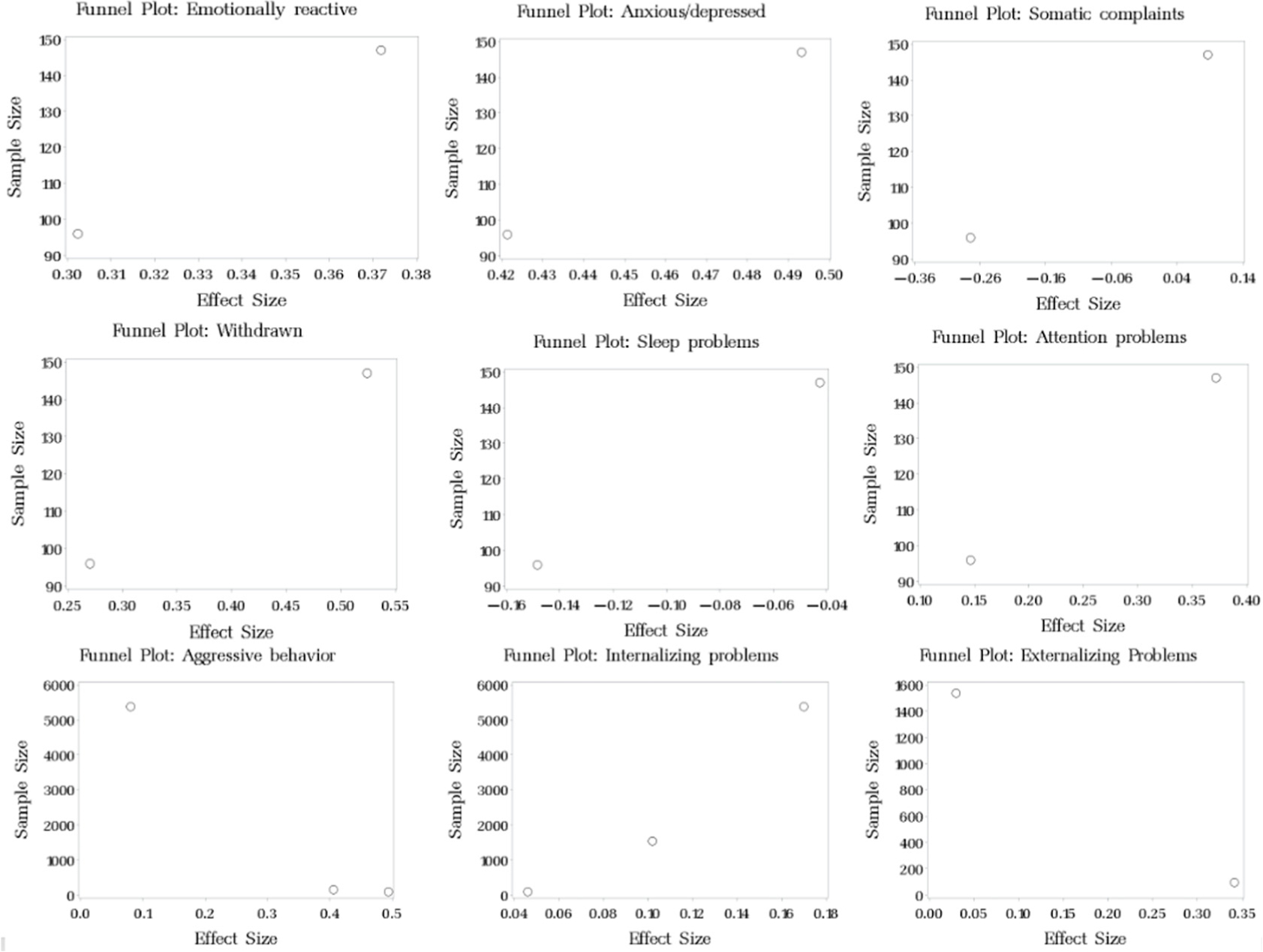
Funnel plot of studies examining changes in CBCL pre- and post-pandemic.

The effect sizes for the multiple subscales of CBCL (Figure 4) showed evidence for small increases in emotional reactivity (d = 0.344, 95% CI 0.087 to 0.6), anxiety/depression (d = 0.464, 95% CI 0.263 to 0.722), withdrawal symptoms (d = 0.42, 95% CI 0.163 to 0.678), attention problems (d = 0.28, 95% CI 0.024 to 0.536), and aggressive behaviors (d = 0.277, 95% CI −0.016 to 0.571, *p* = 0.206), associated with minimal decreases in somatic complaints (d = −0.075, 95% CI −0.428 to 0.277) and sleep problems (d = −0.085, 95% CI −0.340 to 0.169) (Table 2). Internalizing problems increased significantly (d = 0.156, 95% confidence intervals: 0.108, 0.203), while other changes were not significant. Publication bias was not significant (Table A6, Figure A3, Appendix B). Only the aggressive behavior subscale showed significant heterogeneity based on the χ^2^ homogeneity test (*p* = 0.025, I^2^ = 45.65%). The between-study variance was small for the total CBCL score and all the subscales (Table A7, Appendix B).

### 3.5. Individual Study Findings (Not Eligible for Quantitative Meta-Analyses)

Eight papers were not included in the meta-analysis based on the unique outcomes measured and inability to pool their data with other studies.

Davies et al. reported the language skills of children aged 8–36 months from 189 English-speaking families in the UK. The results showed that receptive vocabulary growth (O-CDI) was greater in children who continued to attend early childhood education and care (ECEC) over a 6-month period, with stronger positive effects for children from less advantaged backgrounds. Cognitive/executive functions (Early Executive Functions Questionnaire) were boosted by ECEC attendance, regardless of socioeconomic background [52].

Ferrari et al. used Griffiths Scales of Child Development (GSCD) to assess the global development of infants aged 6 and 12 months. The global development scores decreased during the periods of COVID restriction in Italy (pre-*COVID n* = 34, median 98, IQR 97–103 vs. post-*COVID n* = 70, median 94, IQR 90–100; *p* < 0.001), related to the severity of the restrictions [69]. Wermelinger et al. studied child development, specifically evaluating changes in child facial recognition and emotion labeling. No pandemic-related differences occurred in the children’s emotion labeling, suggesting that they received enough input from masked faces to support the normal development of emotion recognition [72].

Fitzpatrick et al. evaluated children for changes in their screen time and temperament utilizing the Children’s Behavior Questionnaire from Spring 2020 *(n* = 316) to Spring 2021 (*n* = 265). Lower parental education contributed to more child screen time at the ages of 3.5 (ß = 1.37, *p* < 0.001) and 4.5 years (ß = 1.76, *p* < 0.001). Based on the cross-lagged panel model, child screen time at age 3.5 significantly contributed to decreased effortful control scores at age 4.5 (ß = −0.10, SE = 0.042; *p* = 0.023), whereas effortful control at age 3.5 did not contribute to child screen time at age 4.5 (ß = 0.016, SE = 0.046; *p* = 0.729) [54,75].

Kolcakoglu et al. utilized the Spence Preschool Anxiety Scale (SPAS) to assess 405 children over a 2-month period during the first lockdown in Turkey and found increases in separation anxiety and physical injury anxiety, associated with increased tantrums (*p* = 0.010), crying attacks (*p* = 0.010), and aggression (*p* = 0.010) in these children during COVID as compared to pre-COVID [56]. Kostev et al. also reported similar trends with increased new anxiety and depression diagnoses from outpatient pediatric practices in Germany during the pandemic, as compared to 2019–2020 [73].

Alonso-Martinez et al. used the Child Self-Regulation and Behavior Questionnaire (CSBQ) to evaluate child self-regulation, as well as internalizing and externalizing problems, in Spain. Over a 6-month period, the children showed significant decreases in physical activity (−43.3 min/day, 95% CI −68.1 to −18.5) and sleep efficiency (−2.09%, 95% CI −4.12 to −0.05), and increases in sedentary time (+50.2 min/day, 95% CI 17.1 to 83.3), associated with greater internalizing (0.17, 95% CI 0.06 to 0.28) and externalizing (0.33, 95% CI 0.23 to 0.44) problems [60]. Hanno et al. reported similar results, showing that internalizing, externalizing, and dysregulated behaviors increased in US children while their adaptive behaviors declined after the pandemic lockdown [74].

## 4. Discussion

To our knowledge, this is the first meta-analysis to characterize the effects of the COVID-19 pandemic on the mental health and developmental outcomes of children aged 0–6 years. Over 3000 papers were screened, yielding 28 studies for systematic review and 20 studies for meta-analyses, which included 22,348 participants from around the world. Like the meta-analyses performed on older children and adolescents [76–78], our findings suggest negative trends in mental health, cognitive, behavioral, and other developmental domains during the COVID-19 pandemic, as compared to pre-pandemic periods. However, many of these effects were not significant. The child behaviors included difficulties in emotional control (SDQ/CBCL data, others [55]), behavioral conduct, and socializing behaviors (SDQ, ASQ-3, CBCL data). Cognitive or executive functions [51,53,71] and overall development also showed some decline (SDQ data, others [68]), and were associated with increases in anxiety, depression, or withdrawal (CBCL data, others [55,72]) and internalizing or externalizing behaviors (CBCL data, others [59,73]) during the pandemic. Because of the significant heterogeneity and small numbers of studies using the same instrument, most of the findings from our meta-analyses were not significant, except for the internalizing problems from the CBCL data. Despite this, the patterns of similar findings across all the instruments raise concern for early childhood development during the pandemic.

The SDQ assessments showed negligible negative effects on emotional symptoms, conduct problems, and prosocial behaviors, whereas the CBCL data showed a small negative effect on emotional reactivity, anxiety/depression, and withdrawal. Small negative effects were also seen with attention problems, aggressive behaviors, and internalizing and externalizing problems, all of which are consistent with the current literature on the effects of stress in children [79–81]. These results can be explained by studies that show that prolonged stress can disrupt brain circuitry and stress regulatory systems that can affect physiology and behavior [82–84].

In addition to behavioral and mental health, our meta-analysis also analyzed changes in child development on the ASQ-3. Overall, the effect sizes for all ASQ-3 domains were small, suggesting minimal negative effects of the pandemic on fine motor skills and personal–social skills, whereas communication, gross motor skills, problem solving, and total scores showed small improvements. Interestingly, Hessami et al. reported contrasting findings from a systematic review of infant neurodevelopment, with increased risks for communication delay among infants exposed to COVID-19 pandemic lockdowns in the first year of life [85]. Infancy and early childhood are sensitive periods for cognitive and language development, in which negative experiences can undermine a child’s typical developmental trajectory [86]. Davies et al. also found lower language skills among children not receiving early educational enrichment during the pandemic [53]. Our meta-analysis for ASQ-3 data included only three studies, which could not capture the full impact of the pandemic on early neurodevelopment.

Our study showed small effects on various child mental and developmental health domains in a limited sample size. Further research is needed and may find, with a larger sample size, that these issues are more extensive than our study has been able to capture. These small changes can greatly affect a child’s future, and continuing to spurn these issues based on lack of statistical significance may further contribute to the growing mental health crisis across the globe. Our findings, and those of other studies analyzing mental health in children, bring attention to the need for resources and interventions to address the effects of the pandemic [38]. These interventions, whether community-based or primary-care driven, should focus on strengthening family social supports and positive parenting techniques, and teach parents how to facilitate social, emotional, and language skills [31–33,86–88]. In addition to improving access to programs that support families, research evaluating these programs and their efficacy is also needed [88]. Finally, more widespread screening for development and behavioral issues should be implemented to better characterize the full extent of the effect of the COVID-19 pandemic on early childhood development [72].

There are limitations to the current study. The findings from the quantitative meta-analyses did not reach significance because of the heterogeneity of the study designs, children’s characteristics, timing of assessments, small sample sizes, variable durations between pre- and post-pandemic evaluations, and inconsistent/incomplete application of the chosen tools. In addition, assessment methods such as the SDQ, ASQ-3, and CBCL are mainly designed as screening tools rather than diagnostic instruments. Therefore, they were not sensitive enough to reveal subtle changes in the children’s development. Some studies using the SDQ, CBCL, and ASQ-3 presented their results in a way that did not allow the calculation of pre- vs. post-pandemic effect sizes. Some studies also did not use all the available subscales in these screening methods, thus limiting the data available from the subscales. Given the limited data, only one reviewer performed data extraction. However, these data and the articles were then subsequently reviewed by three additional reviewers to ensure the accuracy of the extraction.

To be eligible, all the studies were required to evaluate patients at two time points. However, the interval time between the two evaluations ranged from 3 weeks to 12 months. This discrepancy across studies may have contributed to the vast differences in the study results. Additionally, there was a lack of diversity in patient representation; specifically, few studies looked at patients with neurodevelopmental or psychiatric diagnoses. Additionally, language bias may have been present, as only articles available in English were able to be included. A large majority of the studies utilized parent evaluations and reports of the symptoms of the child, which may have led to reporting bias and higher reporting of problem behaviors.

Additionally, this meta-analysis and systematic review did not evaluate the intrinsic familial protective factors or assistance programs instituted in various countries during the pandemic. Various countries instituted assistance programs throughout the pandemic to support families during the pandemic and quarantine periods, which may have also contributed to the small effects seen in this study.

## 5. Conclusions

This systematic review and meta-analysis of longitudinal cohorts suggests that pandemic-related stressors and social restriction mandates were disruptive for the development and behavioral health of young children across the world. While the effect sizes in our study were small, it is important to consider that even these small effects can make a significant difference in a child’s life course trajectory by further escalating pre-existing developmental concerns or mental health conditions, as well as adding to the cohorts of children having such conditions. These results identify a strong need for standardized early childhood assessment and intervention via child-supportive services, community-based resources, and family-resilience-building programs to address the risk of mental health problems among school children, adolescents, and youth. This evidence provides a strong impetus for policymakers’ consideration of measures to safeguard proactive childhood policies. It is critical that we act now to address these issues and develop a more robust infrastructure to support families in the event of another global pandemic.

## Supplementary Material

ISSM MOOSE Checklist

PRISMA Checklist

## Figures and Tables

**Table 1.** Study characteristics of articles included in the systematic review and meta-analysis.

Reference (First Author, Year)	Country, Study Design	Sample Size (*n*)	Age Range	Outcomes	Evaluation Methods	Time Points Evaluated	Study Quality
Laurie Miller, 2022 [48]	Nepal, Longitudinal Study	368	6–66 Months (mo)	Ages & Stages Questionnaire (ASQ-3)	Mixed-effects regression model	December 2019, September 2021	Good (11)
Elisa Di Giorgio, 2021 [49]	Italy, Retrospective cohort study	245	2–6 years (yrs)	Strengths & Difficulties Questionnaire (SDQ)	McNemar’s test, Repeated measure ANOVA, Tukey HSD test, Pearson correlations, multiple linear regression, Durbin-Watson test	February 2020, April 2020	Good (10)
Alessandra Frigerio, 2022 [50]	Italy, Longitudinal study	74	1.5–5 yrs	Child Behavior Checklist (CBCL)	Hierarchical linear models	April 2018, June 2020	Good (11)
Annie Imboden, 2021 [51]	US, Prospective cohort study	1024	6–36 mo	ASQ-3	Mann-Whitney U test, Kruskal-Wallis test	October 2018-January 2019; October 2020-January 2021	Good (9)
Catherine Davies, 2021 [52]	UK, Prospective cohort study	189	8–36 mo	Child Depression Inventory (O-CDI), Early Executive Function Questionnaire (EEFQ)	Structural equation modeling, multiple linear regression analyses	November-December 2020, June 2021	Good (11)
Ina Specht, 2021 [53]	Denmark, Prospective cohort study	40	2–4 yrs	SDQ	Paired *t* test, general linear models	February 2020, April 2020	Good (10)
Caroline Fitzpatrick, 2022 [54]	Canada, Longitudinal study	316	3–5 yrs	CBCL-Short form	Cross-lagged panel model	April 2020, April 2021	Good (9)
Chiara Cantiani, 2021 [55]	Italy, Longitudinal study	188	2–6 yrs	CBCL,	Independent-sample *t* test, Welch test, Pearson chi-squared	February 2020, May 2020	Good (11)
Keyser Kolcakoglu, 2021 [56]	Turkey, Prospective cohort study	405	3–7 yrs	Preschool Anxiety Scale (PAS)	McNemar-Bowker test, one-sample t test analysis, post hoc Šídák pairwise comparison test	April 2020, June 2020	Good (9)
Xiuxiu Ding, 2022 [57]	China, Longitudinal study	1595	3–6 yrs	SDQ	Pearson’s chi-squared test, paired-sample *t* test, multivariable linear regressions	September 2019, January 2021	Good (13)
Kim Hyunshik, 2021 [58]	Japan, Longitudinal study	301	3–5 yrs	SDQ	Paired *t* test	October 2019, October 2020	Good (9)
Avigail Gordon-Hacker, 2022 [59]	Israel, Prospective cohort study	230	2–5 yrs	SDQ	MCAR test, multilevel models	September 2020, March 2021	Good (11
Alicia Alonso-Martínez, 2021 [60]	Spain, Prospective cohort study	268	4–6 yrs	Child Self-regulation & Behavior Questionnaite (CSBQ)	Analysis of covariance, Shapiro-Wilk test, and Levene’s test	September 2019, April 2020	Good (10)
Anna Wenter, 2022 [61]	Austria, Italy, Retro- and prospective cohort study	951	3–6 yrs	CBCL	Regression model, Bonferroni correlation, multilevel modeling	April 2020, January 2021, Jul 2021, January 2022	Good (11)
Noa Gueron-Sela, 2023 [62]	Israel, Longitudinal study	313	2–5 yrs	SDQ	MANOVA, Bayesian regression analyses	March 2020, October 2020, January 2021, March 2021	Good (12)
Chen Huan Ma, 2022 [63]	China, Cross-sectional comparison	2110	3–6 yrs	SDQ	Chi-squared test, *t* test, logistic regression analyses	March 2022, May 2022	Good (10)
Joseph Murray, 2023 [64]	Brazil, Prospective cohort study	2083	1–4 yrs	SDQ,	Dependent *t* tests, linear regression modeling	September 2019, September 2020	Good (12)
Manuela Gulde, 2022 [65]	Germany, Longitudinal study	158	3–36 mo	SDQ,	Multiple linear regressions	2013–2017, July 2020, May 2021	Good (10)
Hiromichi Hagihara, 2022 [66]	Japan, Prospective cohort study	253	0–6 yrs	SDQ	Linear mixed modeling	April 2020, November 2020, February 2021	Good (9)
Eugenia Conti, 2020 [67]	Italy, Longitudinal study	61	1.5–5 yrs	CBCL	T score comparison, multiple linear regression analyses	September 2019, May 2020	Good (10)
Stephanie Thompson, 2022 [68]	US, Longitudinal study	147	6–36 mo	CBCL	Repeated measures ANOVA	April 2020, October 2020	Good (12)
Eleonora Ferrari, 2022 [69]	Italy, Prospective cohort study	104	3–36 mo	Griffiths Scales of Child Development (GSCD)	Chi-squared, Mann-Whitney U test	March 2019, April 2021	Good (12)
Peiyuan Huang, 2021 [70]	China, Prospective cohort study	2500	6–12 mo	ASQ3	Chi-squared test, log-binomial regression	January 2020, March 2020	Good (11)
Sumayya Saleem, 2022 [71]	Canada, Longitudinal study	179	1–4 yrs	SDQ	Chi-squared test of independence, latent profile analyses with gamma distribution	2016, November 2020	Good (11)
Stephanie Wermelinger, 2022 [72]	Switzerland, Cross-sectional study	30	4–6 years	The Child Faces Task, Children’s Social Understanding Scale	Mixed model	2019, November 2021	Fair (7)
Karel Kostev, 2021 [73]	Germany, Retrospective cross-sectional study	200,600	2–5 years	New anxiety/depression diagnoses	Chi-squared test	April 2019, December 2020	Fair (7)
Emily Hanno, 2021 [74]	US, Longitudinal study	2880	3–6 years	Behavior Assessment System for Children Behavioral and Emotional Screening System (BRIEF-P)	Fixed-effect analyses	2017, 2019, Post-March 2020	Good (10)
Seyma Çiçek, 2021 [75]	Turkey, Two-period descriptive study	346	2–6 years	SDQ	Chi-squared test, independent-sample *t* test	February 2019, June 2021	Good (9)

**Table 2.** Meta-analyses of child assessments pre- and post-pandemic.

SDQ and Subscales	Effect Size	95% CI
Emotional	0.07	(−0.044, 0.184)
Peer Problem	−0.112	(−0.275, 0.501)
Conduct	0.081	(−0.111, 0.273)
Hyperactivity	−0.012	(−0.124, 0.1)
Prosocial Behavior	−0.145	(−0.474, 0.185)
Total SDQ Score	−0.088	(−0.247, 0.07)
ASQ-3 and Subscales		
Communication	0.274	(−0.08, 0.628)
Gross Motor	0.111	(−0.127, 0.349)
Fine Motor	−0.036	(−0.384, 0.312)
Personal-Social	−0.036	(−0.215, 0.143)
Problem-Solving	0.065	(−0.041, 0.171)
Total ASQ-3 Score	0.069	(−0.032, 0.17)
CBCL and Subscales		
Emotionally Reactive	0.344	(0.087, 0.6)
Anxious/Depressed	0.464	(0.263, 0.722)
Somatic Complaints	−0.075	(−0.428, 0.277)
Withdrawn	0.42	(0.163, 0.678)
Sleep Problems	−0.085	(−0.34, 0.169)
Attention Problems	0.28	(0.024, 0.536)
Aggressive Behavior	0.277	(−0.016, 0.571)
Internalizing Problems	0.156	(0.108, 0.203)
Externalizing Problems	0.123	(−0.156, 0.402)

## Data Availability

The original contributions presented in this study are included in the article/Supplementary Materials. Further inquiries can be directed to the corresponding author/s.

## Appendix A

MeSH terms used in systematic review

PubMed (2456)

(“Child, Preschool”[Mesh] OR Preschool* [tw] OR pre-school* [tw] OR infant*[tw] OR baby[tw] OR babies[tw] OR toddler[tw] OR toddlers[tw] OR “school aged”[tw] OR “early child*” OR “early age*” OR “early life” OR “elementary school*”[tw] OR infant[Mesh:noexp]) AND (“covid 19” [tw] OR covid [tw] OR covid19 [tw] OR “ncov 2019” [tw] OR “novel coronavirus” [tw] OR “sars cov 2” [tw] OR “sars cov-2” [tw] OR “ncov 2019” [tw] OR sarscov2 [tw] OR (wuhan [tw] AND coronavirus* [tw]) OR “corona virus*” [tw] OR “coronavirus disease 2019” [tw] OR “coronavirus disease 19” [tw] OR “2019 ncov” [tw] OR 2019nCoV [tw] OR “coronavirus 2” [tw] OR “Coronavirus”[Mesh:NoExp] OR “SARS-CoV-2”[Mesh] OR “COVID-19 Testing”[Mesh] OR “COVID-19” [mesh] OR “COVID-19 Vaccines”[Mesh] OR “Receptors, Coronavirus”[Mesh] OR “Spike Glycoprotein, Coronavirus”[Mesh] OR “SARS-CoV-2 variants” [Supplementary Concept] OR pandemic[tw] OR “social isolation”[tw] OR quarantin*[tw] OR lockdown*[tw] OR “social distance”[tw]) AND (attachment[tiab] OR “attachment theor*”[tw] OR “life span theor*”[tw] OR “parent child synchrony”[tw] OR “parent child bonding*”[tw] OR “social bonding*”[tw] OR “maternal child bonding*”[tw] OR “mother child bonding*”[tw] OR “father child bonding*”[tw] OR “paternal child bonding*”[tw] OR “paternal bond”[tiab:~4] OR “maternal bond”[tiab:~4] OR “paternal bonding”[tiab:~4] OR “maternall bonding”[tiab:~4] OR “father bond”[tiab:~4] OR “mother bond”[tiab:~4] OR “family relations”[mesh] OR “socioeconomic advers*”[tw] OR adversit*[tiab] OR “child development”[mesh] OR “language development”[mesh] OR “child behavior”[mesh] OR “mental disorders”[mesh] OR “social behavior disorders”[Mesh] OR “pediatric obesity”[mesh] OR “mental health”[mesh] OR “child health”[mesh] OR “psychology, child”[mesh] OR neurodevelop*[tw] OR “psychosocial”[tw] OR “adverse childhood experience*”[tw] OR “early life trauma*”[tw] OR “early life stress”[tw] OR “stress, psychological”[mesh] OR “domestic violence”[tw] OR “Intimate Partner Violence”[tw] OR “student dropouts”[mesh] OR “learning”[mesh] OR “underachievement”[mesh] OR “Stress Disorders, Traumatic”[mesh] OR ((mental[tiab] OR “motor skills”[mesh] OR “communication”[tw] OR psycho*[tiab] OR neuro*[tiab] OR motor[tiab] OR verbal[tiab] OR cogniti*[tiab] OR behavior*[tiab] OR emotion*[tiab] OR impact[tiab] OR academic[tw]) AND (development*[tw] OR “long term”[tiab] OR consequence[tiab] OR sequel*[tiab] OR “grow”[tiab] OR growth[tiab] OR disorder*[tw] OR outcome*[tiab] OR problem*[tiab]))) AND 2018:2022[DP] AND English[la]

Embase (2316)

(‘preschool child’/exp OR preschool*:ti,ab,kw OR ‘pre school*’:ti,ab,kw OR toddler:ti,ab,kw OR toddlers:ti,ab,kw OR ‘toddler’/exp OR ‘school aged’:ti,ab,kw OR ‘early child*’ OR ‘early age*’ OR ‘early life’ OR ‘elementary school*’:ti,ab,kw OR infant*:ti,ab,kw OR baby:ti,ab,kw OR babies:ti,ab,kw OR ‘infant’/de OR ‘baby’/exp OR ‘high risk infant’/exp OR ‘hospitalized infant’/exp) AND (‘covid 19’:ti,ab,kw OR covid:ti,ab,kw OR covid19:ti,ab,kw OR ‘novel coronavirus’:ti,ab,kw OR ‘sars cov 2’:ti,ab,kw OR ‘sars cov-2’:ti,ab,kw OR ‘ncov 2019’:ti,ab,kw OR sarscov2:ti,ab,kw OR (wuhan:ti,ab,kw AND coronavirus*:ti,ab,kw) OR ‘corona virus*’:ti,ab,kw OR ‘coronavirus disease 2019’:ti,ab,kw OR ‘coronavirus disease 19’:ti,ab,kw OR ‘2019 ncov’:ti,ab,kw OR 2019ncov:ti,ab,kw OR ‘coronavirus 2’:ti,ab,kw OR ‘coronavirus infection’/de OR ‘coronavirus disease 2019’/exp OR ‘covid-19 testing’/exp OR ‘sars-cov-2 vaccine’/exp OR ‘coronavirus receptor’/exp OR ‘coronavirus spike glycoprotein’/exp OR ‘sars-cov-2’:ti,ab,kw OR pandemic:ti,ab,kw OR ‘social isolation’:ti,ab,kw OR quarantin*:ti,ab,kw OR ‘quarantine’/exp OR lockdown*:ti,ab,kw OR ‘lockdown’/exp OR ‘social distance’:ti,ab,kw OR ‘isolation’/exp) AND (attachment:ti,ab OR ‘attachment theor*’:ti,ab,kw OR ‘life span theor*’:ti,ab,kw OR ‘parent child synchrony’:ti,ab,kw OR ‘parent child bonding*’:ti,ab,kw OR ‘social bonding*’:ti,ab,kw OR ‘maternal child bonding*’:ti,ab,kw OR ‘mother child bonding*’:ti,ab,kw OR ‘father child bonding*’:ti,ab,kw OR ‘paternal child bonding*’:ti,ab,kw OR ((maternal OR paternal OR father OR mother) NEAR/4 bond*):ti,ab,kw OR ‘family relation’/exp OR ‘socioeconomic advers*’:ti,ab,kw OR adversit*:ti,ab OR ‘child development’/exp OR ‘language development’/exp OR ‘psychosocial development’/exp OR ‘speech development’/exp OR ‘child behavior’/exp OR ‘mental disease’/exp OR ‘personality disorders’/exp OR ‘childhood obesity’/exp OR ‘childhood trauma’/exp OR ‘mental health’/exp OR ‘child health’/exp OR ‘child psychology’/exp OR ‘cognitive psychology’/exp OR neurodevelop*:ti,ab,kw OR ‘psychosocial’:ti,ab,kw OR ‘childhood adversity’/exp OR ‘childhood stress’:ti,ab,kw OR ‘adverse childhood experience*’:ti,ab,kw OR ‘early life trauma*’:ti,ab,kw OR ‘early life stress’:ti,ab,kw OR ‘mental stress’/exp OR ‘domestic violence’/exp OR ‘domestic violen*’:ti,ab,kw OR ‘intimate partner violence’:ti,ab,kw OR ‘school dropout’/exp OR ‘student* near/6 dropout*’:ti,ab,kw OR ‘learning’/exp OR ‘achievement’/exp OR ‘anxiety disorder’/exp OR (((mental OR motor OR movement OR speech OR ‘communication’ OR psycho* OR neuro* OR verbal OR cogniti* OR behavior* OR emotion* OR impact OR academic) NEAR/15 (development* OR ‘long term’ OR consequence OR sequel* OR ‘grow’ OR growth OR disorder* OR outcome* OR problem* OR dysfunction*)):ti,ab,kw)) AND [2018–2023]/py AND ([article]/lim OR [article in press]/lim OR [data papers]/lim OR [editorial]/lim OR [erratum]/lim OR [letter]/lim OR [note]/lim OR [review]/lim OR [short survey]/lim) AND [english]/lim PsycINFO (OVID) (621)

1. “Child, Preschool”.mh. or Preschool*.mp. or pre-school*.mp. or infant*.mp. or baby.mp. or babies.mp. or toddler.mp. or toddlers.mp. or “school aged”.mp. or “early child*”.mp. or “early age*”.mp. or “early life”.mp. or “elementary school*”.mp. or exp Preschool Students/OR exp Nursery school students/OR exp kindergarten students/(342251)
2. (“covid 19” or covid or covid19 or “ncov 2019” or “novel coronavirus” or “sars cov 2” or “sars cov-2” or “ncov 2019” or sarscov2 or “corona virus*” or “coronavirus disease 2019” or “coronavirus disease 19” or “2019 ncov” or 2019nCoV or “coronavirus 2”).mp. or “Coronavirus”.mh. or “SARS-CoV-2”.mh. or “COVID-19 Testing”.mh. or “COVID-19”.mh. or “COVID-19 Vaccines”.mh. or “Receptors, Coronavirus”.mh. or “Spike Glycoprotein, Coronavirus”.mh. or “SARS-CoV-2 variants”.mp. or pandemic.mp. or “social isolation”.mp. or quarantin*.mp. or lockdown*.mp. or “social distance”.mp. or exp COVID-19/or exp pandemics/or (wuhan and coronavirus*).mp. (49935)
3. attachment.ti,ab. OR “attachment theor*”.mp. OR “life span theor*”.mp. OR “parent child synchrony”.mp. OR “parent child bonding*”.mp. OR “social bonding*”.mp. OR “maternal child bonding*”.mp. OR “mother child bonding*”.mp. OR “father child bonding*”.mp. OR “paternal child bonding*”.mp. OR ((father OR paternal OR mother OR maternal) ADJ4 bond*) OR “family relations”.mh. OR “socioeconomic advers*”.mp. OR adversit*.ti,ab. or exp attachment theory/or exp parent child relations/OR exp separation anxiety/OR exp domestic violence/OR (“child development” or “language development” or “child behavior” or “mental disorders” or “social behavior disorders” or “pediatric obesity” or “mental health” or “child health” or “psychology, child” or “stress, psychological” or “student dropouts” or learning or “underachievement” or “Stress Disorders, Traumatic”).mh. or (neurodevelop* or “psychosocial” or “adverse childhood experience*” or “early life trauma*” or “early life stress” or “domestic violence” or “Intimate Partner Violence”).mp. or exp Childhood Development/or exp Psychosexual Development/or exp Psychosocial Development/or exp Neonatal Development/or exp Intellectual Development/or exp Psychomotor Development/or exp Cognitive Development/or exp Psychological Development/or exp Early Childhood Development/or exp Emotional Development/or exp Infant Development/or exp Language Development/or exp Personality Development/or exp school dropouts/or exp Posttraumatic Stress Disorder/or exp Posttraumatic Stress/or exp “Stress and Trauma Related Disorders”/or exp Psychological Stress/or exp child health/or exp academic underachievement/or exp mental health/or exp mental disorders/or exp behavior disorders/or exp Childhood Adversity/or ((mental.ti,ab. or “motor skills”.mh. or “communication”.mp. or psycho*.ti,ab. or neuro*.ti,ab. or motor.ti,ab. or verbal.ti,ab. or cogniti*.ti,ab. or behavior*.ti,ab. or emotion*.ti,ab. or impact.ti,ab. or academic.mp.) and (development*.mp. or “long term”.ti,ab. or consequence.ti,ab. or sequel*.ti,ab. or “grow”.ti,ab. or growth.ti,ab. or disorder*.mp. or outcome*.ti,ab. or problem*.ti,ab.)) (2373046)
4. english.lg. (4881748)
5. 1 AND 2 AND 3 AND 4
6. limit 5 to yr = “2018–2023”

## Appendix B

**Table A1.** Full study characteristics table.

Reference (First Author, Year)	Country, Study Design	Sample Size (*n*)	Age Range	Clinical Context	Outcomes	Evaluation Methods	Main Findings	Conclusions	Study Quality
Laurie Miller, 2022 [48]	Nepal, Longitudinal study	368	6–66 months	Low-income subsistence farmers in Nepal	ASQ-3	Mixed-effects regression model	Total ASQ scores did not change. However, communication scores increased, while fine motor and personal-social skills decreased.	Increases in communication may have reflected more exposure to adult language. Decline in other domains may have been due to closure of preschools.	Good (11)
Elisa Di Giorgio, 2021 [49]	Italy, Retrospective cohort study	245	2–6 years	Recruited via research websites and social media groups	SDQ	McNemar’s test, repeated-measures ANOVA, Tukey HSD test, Pearson correlations, multiple linear regression, Durbin-Watson test	Emotional symptoms, lack of discipline, and hyperactivity increased from pre-quarantine to during the quarantine. This increase in symptoms was also associated with changes in maternal sleep and stress.	The Italian lockdown and subsequent closure of schools and home confinement were challenging for mothers and their children.	Good (10)
Alessandra Frigerio, 2022 [50]	Italy, Longitudinal study	74	1.5–5 years	Recruited from ongoing study	CBCL	Hierarchical linear models	Children’s emotional and behavioral problems increased from pre- to during lockdown.	COVID-19 lockdown negatively impacted children’s psychological well being.	Good (11)
Annie Imboden, 2021 [51]	US, Prospective cohort study	1024	6–36 months	Two primary pediatric practices, one rural and one suburban	ASQ-3	Mann-Whitney U test, Kruskal-Wallis test	There were no significant differences in pre- or post-pandemic total ASQ3 scores. When separated by age and domain, there was a decrease in communication scores in some age groups.	Given the decrease in communication scores, efforts should be directed in making face-to-face interactions safer.	Good (9)
Catherine Davies, 2021 [52]	UK, Prospective cohort study	189	8–36 months	Recruited via research websites and social media groups	O-CDI, EEFQ	Structural equation modeling, multiple linear regression analyses	Language and executive function for children from low SES were disproportionately affected by lockdown due to low ECEC access.	During lockdown, ECECs should be kept open, especially to those populations that are less advantaged.	Good (11)
Ina Specht, 2021 [53]	Denmark, Prospective cohort study	40	2–4 years	Randomly selected parents from 3 kindergartens	SDQ	Paired *t* test, general linear models	Increase in total score, hyperactivity, prosocial behavior, and externalizing behavior.	Tendency towards adverse consequences in child emotional-behavioral function in relation to home confinement.	Good (10)
Caroline Fitzpatrick, 2022 [54]	Canada, Longitudinal study	316	3–5 years	Recruited in multiple settings	Children’s Behavior Questionnaire-Short form	Cross-lagged panel model	Higher levels of screen time during the pandemic are associated with lower levels of effortful control.	In the context of increasing media use during the pandemic, parents should be encouraged to engage in play and establish a family media plan.	Good (9)
Chiara Cantiani, 2021 [55]	Italy, Longitudinal study	188	2–6 years	Recruited from general population	CBCL,	Independent-sample *t* test, Welch test, Pearson chi-squared	There was an increase in anxiety, depression, and externalizing problems during the COVID-19 pandemic across neurotypical children and those at high risk for neurodevelopmental delay.	The COVID-19 pandemic had a negative impact on the emotional and behavioral profiles of Italian preschoolers, irrespective of neurodevelopmental delay risk factors.	Good (11)
Keyser Kolcakoglu, 2021 [56]	Turkey, Prospective cohort study	405	3–7 years	Snowball sampling via WhatsApp distribution	Spence Preschool Anxiety Scale	McNemar-Bowker test, one-sample *t* test analysis, post hoc Šídák pairwise comparison test	The total SPAS anxiety scores and emotional and behavioral changes were higher during COVID compared to pre-COVID.	Tantrums and crying attacks increased in children during Turkey’s curfew.	Good (9)
Xiuxiu Ding, 2022 [57]	China, Longitudinal study	1595	3–6 years	Convenience sampling of children in preschools	SDQ	Pearson’s chi-squared test, paired-sample *t* test, multivariable linear regressions	Those who experienced severe impact of COVID-19 had more sleep problems, increased anxiety, and problematic behaviors.	The severe impact of COVID-19 increased the risk for anxiety symptoms and problematic behaviors in preschool children.	Good (13)
Kim Hyunshik, 2021 [58]	Japan, Longitudinal study	301	3–5 years	Convenience sampling of childcare centers	SDQ	Paired *t* test	Prosocial behavior decreased and hyperactivity increased during the pandemic. Physical activity decreased and sedentary behavior increased.	The adverse effects of COVID-19 on mental health suggest the need for strategies to improve physical activity and prevent long-term health risks.	Good (9)
Avigail Gordon-Hacker, 2022 [59]	Israel, Prospective cohort study	230	2–5 years	Recruited via online research platform	SDQ	MCAR test, multilevel models	Increase in maternal and child symptomatology during the lockdowns and a decrease at the end of lockdown.	Lockdowns had adverse effects on mothers and children.	Good (11
Alicia Alonso-Martínez, 2021 [60]	Spain, Prospective cohort study	268	4–6 years	Recruited from among preschoolers	CSBQ	Analysis of covariance, Shapiro-Wilk test, and Levene’s test	Preschoolers showed a significant increase in internalizing and externalizing problems during the lockdown.	Covid-19 negatively affected physical activity, sedentary behavior, sleep, and self regulation of preschoolers.	Good (10)
Anna Wenter, 2022 [61]	Austria, Italy, Retro- and prospective cohort study	951	3–6 years	Schools in Tyrolean COVID hotspots	CBCL	Regression model, Bonferroni correlation, multilevel modeling	There was an increase in internalizing problems pre- and post-lockdown in preschool children.	Targeted support for vulnerable children may prevent the longer-term development of psychopathologies and contribute to psychosocial resilience in society.	Good (11)
Noa Gueron-Sela, 2023 [62]	Israel, Longitudinal study	313	2–5 years	Recruited via online research platform	SDQ	MANOVA, Bayesian regression analyses	Children’s conduct and emotional problems increased from pre- to post-lockdown. Media use was positively correlated with emotional and conduct problems.	Media use may be meeting important needs for parents and children in the short term, and children’s behavior changes may return to more typical patterns.	Good (12)
Chen Huan Ma, 2022 [63]	China, Cross-sectional comparison	2110	3–6 years	Cluster sampling method utilizing random selection from seven districts	SDQ	Chi-squared test, *t* test, logistic regression analyses	When compared to 2019, children experiencing the COVID lockdown had fewer emotional symptoms, but higher levels of conduct problems, total SDQ, peer problem scores, and prosocial behavior scores.	There was an increase in the frequency of emotional and behavioral problems, especially regarding conduct problems and peer problems, in preschool children during the lockdown.	Good (10)
Joseph Murray, 2023 [64]	Brazil, Prospective cohort study	2083	1–4 years	Mothers recruited at birth as part of the Pelotas Birth Cohort Study	SDQ,	Dependent *t* tests, linear regression modeling	Child conduct problems and hyperactivity decreased. However, mean levels of emotional problems were similar before and during the pandemic. Children in families with lower income before the pandemic showed increases in all types of mental health problems during the pandemic.	The impact of the COVID-19 pandemic on mental health presented a mixed picture, but for families in poverty, material and interpersonal difficulties were associated with increases in mental health problems among children.	Good (12)
Manuela Gulde, 2022 [65]	Germany, Longitudinal study	158	3–36 months	Mother-child dyads recruited from maternity unit	SDQ,	Multiple linear regressions	Harmful parental behavior also correlated significantly with lack of coping strategies, hyperactivity, emotional problems, and externalizing problems.	Maternal attachment and the associated coping skills and corresponding parental behavior significantly influenced children’s mental health during the pandemic.	Good (10)
Hiromichi Hagihara, 2022 [66]	Japan, Prospective cohort study	253	0–6 years	Randomly selected from an online database	SDQ	Linear mixed modeling	Preschoolers seemed to exhibit more problematic social behaviors during lockdown, as opposed to when schools were open at T3.	Changes in children’s perceived proximity to others can negatively influence their social development and well-being, even if this is not serious enough to increase problematic behavior.	Good (9)
Eugenia Conti, 2020 [67]	Italy, Longitudinal study	61	1.5–5 years	Recruited from a specialized hospital for care for neurologic and psychologic disorders	CBCL	T score comparison, multiple linear regression analyses	There was a significant worsening in somatic complaints and DSM-oriented anxiety in children under 5 years of age. Regression models did not show any specific patterns in effects of finances, hours of treatment prior, or age.	Younger children may be more sensitive to spillover stress from parents. Young children seemed less affected by financial hardship, age, and treatment prior to lockdown.	Good (10)
Stephanie Thompson, 2022 [68]	US, Longitudinal study	147	6–36 months	Recruited from hospitals, clinics, and charitable agencies	CBCL	Repeated-measures ANOVA	Child internalizing and externalizing problems did not increase significantly on average pre- or during lockdown.	COVID-19 contextual hardships predicted changes in maternal mental health symptoms and child adjustment.	Good (12)
Eleonora Ferrari, 2022 [69]	Italy, Prospective cohort study	104	3–36 months	Enrolled in a second-level university hospital	GSCD	Chi--squared, Mann-Whitney U test	6-month-old infants showed worsening in the communication and personal-social domains from pre- to during COVID-19. GDS scrores downtrended during the pandemic in infants.	The severity of the restrictions negatively affects infants’ scores, as shown in the results of the linear regression: the more the degree of restriction increases, the more the GDS decreases.	Good (12)
Peiyuan Huang, 2021 [70]	China, Prospective cohort study	2500	6–12 months	Recruited from medical center at time of birth	ASQ3	Chi-squared test, log-binomial regression	Experiencing COVID-19 in 2020 was associated with a higher risk of neurodevelopmental delay in the fine motor and communication domains in 1-year-old children, while no associations are observed for those at 6 months of age.	The COVID-19 pandemic might potentially have a negative impact on child neurodevelopment in specific domains at specific ages, which raises concerns about the development of young children under the COVID-19 pandemic	Good (11)
Sumayya Saleem, 2022 [71]	Canada, Longitudinal study	179	1–4 years	Recruited from City of Toronto subsidy waitlist	SDQ	Chi-squared test of independence, latent profile analyses with gamma distribution	Children with pre-existing high hyperactivity scores showed significant increases in emotional problems, while other groups showed stable or improving mental status.	A substantial proportion of children experienced improvements in mental health following the onset of the COVID-19 pandemic.	Good (11)
Stephanie Wermelinger, 2022 [72]	Switzerland, Cross-sectional study	30	4–6 years	Recruited from database of the research unit at the University of Zurich	The Child Faces Task, Children’s Social Understanding Scale	Mixed model	The results of our study indicate no evidence for pandemic-related differences in social interactions in children’s emotion labeling. Children during the pandemic recognized fare better than children before the pandemic in adult faces.	The COVID-19 pandemic and the according changes in social interactions, such as meeting fewer people, or seeing more people wearing masks, do not substantially relate to preschoolers’ emotion labeling	Fair (7)
Karel Kostev, 2021 [73]	Germany, Retrospective cross-sectional study	200,600	2–5 years	Medical record data from the Disease Analyzer database (IQVIA)	New anxiety/ depression diagnoses	Chi-squared test	The number of children and adolescents with depression and anxiety diagnoses per practice increased in April 2020-December 2020 compared to the same period in 2019. Prevalence of depression and anxiety increased during the pandemic.	It is important to develop more targeted interventions to mitigate the negative impact of the pandemic on child and adolescent mental health.	Fair (7)
Emily Hanno, 2021 [74]	US, Longitudinal study	2880	3–8 years	Data from the Early Learning Study at Harvard (ELS@H)	Behavior Assessment System for Children Behavioral and Emotional Screening System; BRIEF-P	Fixed-effect analyses	Mixed-effects analyses showed children’s externalizing, internalizing, and dysregulated behaviors increased after the shutdown, whereas children’s adaptive behaviors declined.	Children experienced declines in behavioral health and families experienced declines in well-being in the early months of the public health crisis, suggesting the need for family-focused and child-focused policies to mitigate these changes.	Good (10)
Seyma Çiçek, 2021 [75]	Turkey, Two-period descriptive study	346	2–6 years	Children enrolled during admission to hospital setting	SDQ	Chi-squared test, independent-sample *t* test	Total ERC and Emotion Regulation Subscale scores were significantly higher and Lability-Negativity Subscale scores, conduct problems, peer relationship problems, internalizing scores, and total SDQ scores were significantly lower in the pandemic period group than in the pre-pandemic group	ERC and peer relationship problems were lower during the pandemic, regardless of maternal occupation status. The interaction between the pandemic period and the mother’s working status was detected in the prosocial behavior internalization and total difficulties scale.	Good (9)

**Table A2.** Test of publication bias of SDQ.

	Egger Regression		Begg Rank Correlation		Funnel Plot Regression		Publication Bias Present		
Subscale	t Statistic	*p* Value	t Statistic	*p* Value	t Statistic	*p* Value	Left Tail	Right Tail	Both Tails
Emotional Problem	0.747	0.479	−2.502	0.012	−1.245	0.253	No	No	No
Peer Problem	1.609	0.206	−1.445	0.148	−0.609	0.586	No	No	No
Conduct	1.289	0.239	−3.335	0.001	−2.172	0.066	No	No	No
Hyperactivity	2.31	0.069	−3.224	0.001	−1.989	0.103	No	No	No
Prosocial Behavior	−0.882	0.443	−2.119	0.01	1.494	0.232	No	No	No
Total SDQ	2.111	0.125	−2.779	0.01	−1.499	0.231	No	No	No

**Table A3.** Test of homogeneity for SDQ.

Subscale	χ^2^ Homogeneity Test	*p*-Value	Between-Study Variance
Emotional	56.817	<0.0001	0.027
Peer Problem	25.289	<0.0001	0.026
Conduct	171.196	<0.0001	0.078
Hyperactivity	35.644	<0.0001	0.017
Prosocial Behavior	109.692	<0.0001	0.13
Total SDQ	24.003	0.0001	0.024

**Table A4.** Test of publication bias of ASQ-3.

	Egger Regression		Begg Rank Correlation		Funnel Plot Regression		Publication Bias Present		
Subscale	t Statistic	*p* Value	t Statistic	*p* Value	t Statistic	*p* Value	Left Tail	Right Tail	Both Tails
Communication	1.666	0.344	−0.439	0.661	−1.149	0.456	No	No	No
Gross Motor	1.613	0.353	5	<0.0001	−1.247	0.43	No	No	No
Fine Motor	−1.535	0.368	5	<0.0001	1.4	0.395	No	No	No
Personal-Social	−1.503	0.374	6	<0.0002	1.344	0.407	No	No	No
Problem-Solving	n/a	n/a	n/a	n/a	n/a	n/a	n/a	n/a	n/a
Total	n/a	n/a	n/a	n/a	n/a	n/a	n/a	n/a	n/a

n/a: Those statistics are not estimated when there are only two studies included.

**Table A5.** Test of homogeneity for ASQ-3.

Subscale	χ^2^ Homogeneity Test	*p*-Value	Between-Study Variance
Communication	38.186	<0.0001	0.092
Gross Motor	17.452	0.0002	0.039
Fine Motor	37.272	0.3676	0.089
Personal-Social	9.832	0.007	0.02
Problem-Solving	1.074	0.3	0.0005
Total	0.111	0.739	0

**Table A6.** Test of publication bias of CBCL.

	Egger Regression		Begg Rank Correlation		Funnel Plot Regression		Publication Bias Present		
Subscale	t Statistic	*p* Value	t Statistic	*p* Value	t Statistic	*p* Value	Left Tail	Right Tail	Both Tails
Emotionally reactive	n/a	n/a	n/a	n/a	n/a	n/a	n/a	n/a	n/a
Anxious/Depressed	n/a	n/a	n/a	n/a	n/a	n/a	n/a	n/a	n/a
Somatic complaints	n/a	n/a	n/a	n/a	n/a	n/a	n/a	n/a	n/a
Withdrawn	n/a	n/a	n/a	n/a	n/a	n/a	n/a	n/a	n/a
Sleep problems	n/a	n/a	n/a	n/a	n/a	n/a	n/a	n/a	n/a
Attention problems	n/a	n/a	n/a	n/a	n/a	n/a	n/a	n/a	n/a
Aggressive behavior	1.235	0.433	5	0	−1.22	0.437	No	No	No
Internalizing problems	−1	0.5	0.439	0.661	1.201	0.442	No	No	No
Externalizing problems	n/a	n/a	n/a	n/a	n/a	n/a	n/a	n/a	n/a

**Table A7.** Test of homogeneity for CBCL.

Subscale	χ^2^ Homogeneity Test	*p*-Value	Between-Study Variance
Emotionally reactive	0.068	0.794	0
Anxious/Depressed	0.072	0.789	0
Somatic complaints	1.857	0.173	0.03
Withdrawn	0.904	0.342	0
Sleep problems	0.16	0.689	0
Attention problems	0.719	0.4	0
Aggressive behavior	7.36	0.025	0.048
Internalizing problems	1.463	0.481	0
Externalizing problems	2.127	0.145	0.026
